# The Allocation of Vertical Attention in Patients with End-Stage Renal Disease Receiving Dialysis

**DOI:** 10.3390/brainsci11121549

**Published:** 2021-11-23

**Authors:** Aleksandra Mańkowska, Kenneth M. Heilman, Bogdan Biedunkiewicz, Alicja Dębska-Ślizień, John B. Williamson, Michał Harciarek

**Affiliations:** 1 Division of Neuropsychology, Institute of Psychology, University of Gdańsk, 80-309 Gdańsk, Poland; aleksandra.mankowska@ug.edu.pl; 2 North Florida/South Georgia Veterans Affairs Medical Center, Department of Neurology, University of Florida College of Medicine, Gainesville, FL 32608, USA; heilman@neurology.ufl.edu (K.M.H.); john.williamson@ufl.edu (J.B.W.); 3 Brain Rehabilitation Research Center, North Florida/South Georgia Veterans Affairs Medical Center, Gainesville, FL 32608, USA; 4 Department of Nephrology, Transplantology and Internal Medicine, Medical University of Gdańsk, 80-210 Gdańsk, Poland; bbied@gumed.edu.pl (B.B.); adeb@gumed.edu.pl (A.D.-Ś.)

**Keywords:** end-stage renal disease, visual attention, vertical line bisection, ventral and dorsal attentional networks

## Abstract

**Objectives**: Alterations of spatial attention can have adverse effects, such a greater probability of accidents. Patients with end-stage renal disease (ESRD) receiving dialysis have stronger left-sided spatial attentional bias, suggesting that this disorder or treatment alters the brain networks that mediate spatial attention. The hemispheric networks that mediate the allocation of horizontal attention may also influence the allocation of vertical attention. However, the allocation of vertical spatial attention has not been studied in ESRD patients. **Methods**: Twenty-three ESRD patients receiving dialysis and 23 healthy right-handed controls performed line bisections using 24 vertical lines (24 cm long and 2 mm thick) aligned with the intersection of their midsagittal and coronal planes. **Results:** Hemodialyzed ESRD patients had a significantly greater upward bias than healthy controls. The magnitude of this bias was correlated with the duration of the kidney disease. **Conclusions:** The reason why upward attentional bias is increased in hemodialyzed ESRD patients is not known. Further research is needed to better understand the brain mechanism that might account for this bias, as well as its treatment. However, hemodialyzed ESRD patients and their families-caregivers should be made aware of this disorder to avoid accidents such as tripping.

## 1. Introduction

Chronic kidney disease (CKD) is a polysymptomatic illness that results from the progressive degeneration of kidney parenchyma. In the beginning of the CKD, the treatment is usually focused on slowing the progression of the illness. Nonetheless, due to decreasing renal function, patients in stage five of CKD (end-stage renal disease—ESRD) require either kidney transplantation or regular dialysis to treat their uremia. Most dialyzed patients reveal cognitive impairments, including memory disorders, psychomotor slowing, and impaired, sustained visual attention [1,2,3]. Although the exact mechanism accounting for cognitive changes is not entirely known, ESRD patients requiring hemodialysis (hemodialysis—HD) constitute a population that is at high risk of brain infarcts and brain atrophy. This brain atrophy may result from the chronic exposure to toxins associated with the renal failure and/or comorbid conditions that impair cerebral function (e.g., hypertension, diabetes mellitus) [1,4,5,6,7].

Many of the neurocognitive impairments seen in the patients receiving hemodialysis are characterized by deficits in frontal-subcortical cognitive functions, such as executive disorders and psychomotor slowing [3,8,9]. Prior research investigating frontal attentional systems in hemodialyzed patients has also demonstrated that the attentional disorders observed in this group may be relatively selective, predominantly affecting arousal energization that contributes to these patients’ impaired ability to sustain attention [10,11].

When attempting to bisect horizontal lines, healthy alert subjects deviate their bisection toward the left of center, called “pseudoneglect” [12] for review, see Jewell and McCourt, 2000). This deviation in healthy participants is thought to be related to right hemisphere dominance for mediating spatial or global attention [13] required for this task. When compared to healthy control participants, ESRD patients who are being treated with dialysis have an increase in their leftward attentional bias [2]. The reason for this increase in the left-sided bias is not known. However, Denny-Brown and Chambers (1958) posited and provided evidence that, whereas the frontal lobe mediate disengagement, the posterior temporal-parietal lobe mediate engagement–approach, and these two networks exert mutual inhibition. Thus, in the mentioned study [2], we hypothesized that ESRD with dialysis may induce frontal-subcortical dysfunction [3,11,14] that disinhibits the parietal lobes, and with the right parietal lobe being dominant for allocating spatial attention [13,15], there is an enhanced leftward spatial bias.

When attempting to bisect solid vertically oriented lines, normal individuals tend to deviate their bisection upwards [16,17]. There are at least two explanations for the upward bias in line bisections. First, this upward bias may result from the right hemisphere dominance for mediating spatial attention. According to this hypothesis, not only does the right hemisphere mediate leftward attention, but it is also dominant for mediating spatial attention, and therefore induces a leftward bias on horizontal line bisection. In addition, the attentionally dominant right hemisphere may also mediate upward attention and be responsible for the upward bias on vertical line bisection. Support for this postulate comes from Suavansri and colleagues (2012), who have shown that young, healthy adults make higher bisections of vertical lines in the left than right hemispace [17].

The “Landmark Task” is a perceptual-attentional line bisection judgment task that does not require arm–hand movements. Seydell-Greenwald and colleagues (2019) examined neurologically healthy young adults using the landmark test with vertical instead of horizontal stimuli. These investigators found right-lateralized parietal activations similar to those reported in other landmark tests with horizontal lines [18].

After processing by the primary visual cortex, visual stimuli undergo parallel visual processing by two specialized extra-striate areas, a ventral stream located in in the temporal lobes and a dorsal stream located in the parietal lobes [19,20]. Bilateral damage to the ventral stream, as reported by Shelton, Bowers, Duara, and Heilman (1994) and Riddoch (2008) results in the neglect of the upper altitudinal space [21,22]. In contrast, patients with dorsal (parietal cortex) lesions have neglect of lower egocentric space [23]. These observations suggest that the dorsal stream allocates egocentric attention downward, and the ventral stream allocates egocentric attention upward. According to Braddick and coallegues (2003), the majority of neurons in the geniculate that are components of the dorsal stream are magnocellular, and the neurons in the ventral stream are primarily parvocellular. Since the dorsal-magnocellular neurons are larger than ventral-parvocellular neurons, and neurons with larger cell bodies and axon diameters are more susceptible to damage, the dorsal stream may be more susceptible to deterioration then the ventral stream [24,25]. It is therefore possible that dialyzed patients due to uremia and other metabolic disturbances also would have a greater impairment in the dorsal than ventral visual stream, and this imbalance between the dorsal (orienting attention downward) and ventral (orienting upward attention) attentional streams would result in the increase in the upward vertical bias.

To our knowledge, however, it is not currently known if ESRD and dialysis would primarily result in upward or downward deviation in the allocation of vertical spatial attention, and this is the goal of our study. Thus, in this study, we will test two exploratory hypotheses: (1) patients receiving HD will allocate their bisections significantly lower than healthy subjects (right hemisphere hypothesis); (2) patients receiving dialysis will allocate their bisections significantly higher than healthy participants (dorsal hypothesis).

## 2. Methods

### 2.1. Participants

In the present study, we tested a group of 46 right-handed participants. Demographic and clinical data are presented in Table 1.

The biochemical variables (level of creatinine, blood urea nitrogen (BUN), albumin, and hemoglobin (Hb)) are medical markers of kidney functions. The population of patients with ESRD is characterized by elevated levels of creatinine, BUN, and albumin, whereas anemia (according to WHO criteria: Hb < 13 g/dL in men or Hb < 12 g/dL in women) is common in ESRD patients, due in part to reduced erythropoietin production as kidney function declines. Those biochemical variables have been recognized as risk factors for cognitive decline in ESRD patients.

The experimental participants were twenty-three patients (age range: 21–60 years) with ESRD treated with dialysis. To meet the inclusion criteria, each patient had received hemodialysis continuously for a minimum of 6 months (Mean = 17.76 ± 24.76 months). Hemodialysis sessions were conducted 3 times a week, and each lasted for 4–5 h. The testing session always occurred on non-dialysis day after the first dialysis session of the week (e.g., if the patients receive dialysis on Mondays, the testing session was conducted on Tuesday). The inclusion and exclusion criteria included the absence of malignancies, clinically evident cerebrovascular disease, history of psychiatric or neurologic disorders, uncontrolled diabetes, hypertension or anemia, cognitive impairment, learning disabilities, current alcohol or drug abuse, psychoactive medication use, relevant hearing or visual difficulties, or another major organ failure (e.g., cardiac, hepatic, or pulmonary).

In order to match the control with the experimental participants the healthy control participants were recruited after completing recruitment of the patients being treated with dialysis and were subject to the same exclusion criteria as ESRD patients undergoing dialysis. The comparison group consisted of 23 demographically matched controls without a history of kidney disease (glomerular filtration rate ≥90 mL/min/1.73 m^2^), as determined by blood testing conducted ~24 h before the experimental testing.

### 2.2. Apparatus and Procedures

Overall, all procedures were similar to those in our previous study [2] and consisted of an interview, short examination of mood and mental status, and the vertical line bisection task (VLBT) [17,26]. The protocol of the study was approved by the Ethical Committee at the Institute of Psychology, University of Gdańsk. All participants declared general wellbeing on the day of testing and signed an informed consent form. Following experimental procedures, participants have been compensated with 100 PLN (Polish equivalent of ~25 USD). The experimental procedures took place at the Institute of Psychology, University of Gdańsk (Poland).

Additionally, on the day of testing session, the mood of all participants was assessed using the Polish adaptation of Zigmond and Snaith’s hospital anxiety and depression scale (HADS) [27]. The mental status of the participants was assessed using mini mental state examination (MMSE); [28] (see Table 1).

When performing the vertical line bisection task all participants were sitting at a table and were instructed to find the middle of the vertical line (bisect the line) and to mark it. The lines were printed on A4 format paper, were 24 cm long and 2 mm wide, and were sequentially presented on a white, passe-partout frame. This frame with the attached paper with the line was centered at the intersection of the midsagittal and coronal planes. The middle of the line was placed at eye level, and the paper was placed approximately 50 cm (±4 cm) in front of the subject’s chest. The participants used their right hand to make the bisection mark with a pen. After each bisection, the marked sheet of paper was removed and replaced with new clear line, until all 24 bisections were performed. During the task, participants were not given any feedback as to their performance.

The deviations from the midpoint of each line were measured to the nearest millimeter. Upward deviations (above the midpoint) were designated as positive (+ = upward), while downward deviations were designated as negative (− = downward). Finally, the results of 24 bisections for each participant were summed, and an algebraic mean was calculated.

## 3. Results

### 3.1. Statistical Analysis

We used the Statistical Package for the Social Sciences version 26.0 for descriptive statistical analyses. To compare the patients’ performance with that of the control group, we used independent *t*-tests. We set two-tailed statistical significance at *p* < 0.05.

### 3.2. Demographic and Clinical Factors

Overall, both groups did not differ in terms of sex, years of education, and general cognitive status. In addition, the independent *t*-test did not reveal any differences in terms of depression and anxiety symptoms, as measured by the Polish adaptation of the hospital anxiety and depression scale (see Table 1). Additionally, depression did not significantly influence the magnitude of the bias in the group of healthy participants (Pearsons’r = 0.34, *p* = 0.76) or HD patients (Pearsons’r = 0.53, *p* = 0.21)

### 3.3. Vertical Line Bisection Task (VLBT)

One-sample *t*-tests against true midpoint (=0.0, the middle of the line) revealed that both patients receiving hemodialysis (M = 3.35, SD = 3.10); *t*(22) = 5.20, *p ≤* 0.001) and control participants (M = 1.22, SD = 2.88); *t*(22) = 2.03, *p* = 0.05) significantly deviated their bisections upwards.

**Figure 1:**
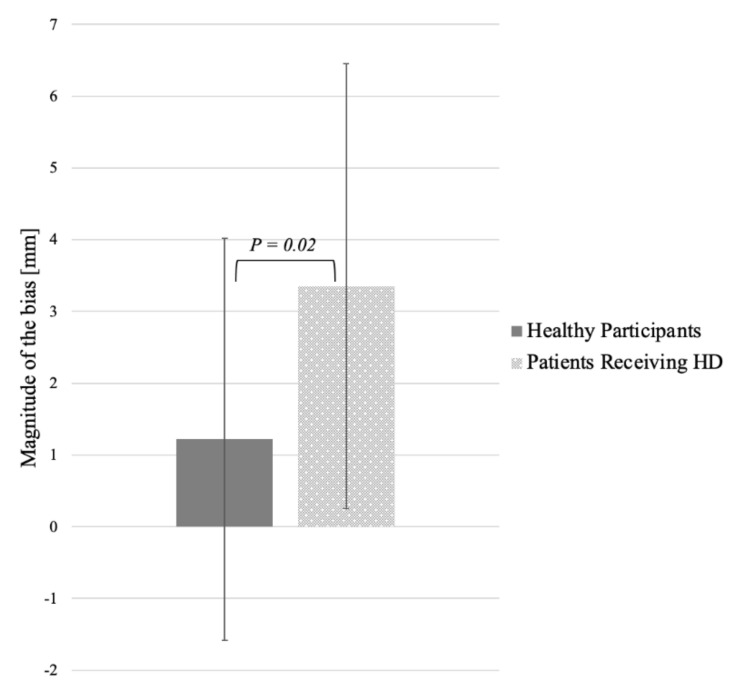
The magnitude of the bias in vertical line bisection task in healthy participants and ESRD patients receiving dialysis.

The results in VLBT of healthy participants (Shapiro–Wilks *W =* 0.97; *p* = 0.69) and HD patients were normally distributed (Shapiro–Wilks *W =* 0.92; *p* = 0.20). Therefore, to learn if there was a group difference in the allocation of spatial attention determined by the performance of a VLBT, an independent *t*-test was conducted. Overall, the analysis revealed that ESRD patients deviated their bisections significantly more upwards than did the control participants, *t*(45) = 4.95, *p* = 0.02, *d =* 0.65 (see Figure 1).

### 3.4. Influence of Medical–Metabolic Factors and Illness Duration on the Vertical Line Bisection Performance

Further, we wanted to learn how medical–metabolic factors and the duration of kidney disease and duration of the dialysis might be related to the bias found in the vertical line bisection test.

Thus, a series of exploratory correlation analysis were performed. Since all variables met the criteria of normal distribution, we used a Pearson’s r. For each group of participants, their hemoglobin, creatinine, urea nitrogen, as well as their disease and dialysis duration and the mean result in VLBT were correlated. The results of this analysis revealed that the magnitude of vertical bias increased with the longer duration (more years) of renal disease (see Table 2).

## 4. Discussion

This study was designed to investigate and compare the direction and magnitude of vertical attentional bias in ESRD hemodialyzed patients and matched controls. With ESRD and hemodialysis, there might be a reduction in hemispheric arousal. Röhl and colleagues reported that this reduction was greatest in temporal lobes, and the temporal lobes have been reported to be important in mediating upward attention; one possible result is that the ESRD hemodialyzed patients would reveal a decrease in the upward vertical bias [29].

Based on the dorsal vulnerability hypothesis [24], our alternative hypothesis was that patients undergoing dialysis would have an impairment of their dorsal attentional visual network. We also hypothesized that HD patients’ dorsal attentional stream might be more susceptible to decline due to hypertension and uremic toxicity. This impairment would result in a greater imbalance in the allocation of spatial attention between the ventral and dorsal attentional streams, and this imbalance would increase in the upward vertical attentional bias. The results of this study revealed that both healthy and hemodialyzed participants have an upward deviation on their attempted vertical bisections. However, the upward bias seen in ESRD receiving hemodialysis was significantly greater than that observed in the healthy adults. Additionally, the upward bias on the line bisection test was positively correlated with the duration of the illness, indicating that the patients with longer duration of the renal failure tend to bisect lines higher than those with a shorter duration. These results appear to support the dorsal vulnerability hypothesis [24,25].

With aging, there is a significant reduction in glomerular filtration rate that can influence cognition in older adults. It has recently been suggested that chronic kidney disease and dialysis may accelerate aging of the central nervous system [6]. Further, we previously used the vertical lines bisections to compare the orientation of vertical attention in younger and older adults. The results of our prior study showed that, although all participants deviated their bisections upwards, older adults allocate their attempted bisections significantly higher. This increase in the upward bias found in older healthy participants is similarly to the hemodialyzed patients in this study [30]. Furthermore, the magnitude of vertical bias was significantly related to the duration of the illness. This result suggests that long-lasting uremia and perhaps cerebrovascular disease, most likely related to chronic hypertension, advances the cerebrum deterioration and accelerates the aging process, as hypothesized by Murray [6].

Denny-Brown and Chambers [31] proposed that, whereas the frontal lobes mediate disengagement and avoidance behaviors, the posterior parietal lobes mediate engagement and approach behaviors. Normally, regarding engagement and disengagement, there is a mutually inhibitory relationship between the frontal lobes and the posterior parietal lobes, and dysfunction to one of these areas will result in a transcortical release of the behaviors mediated by the intact region [31]. Prior research has suggested that patients with ESRD have a decline in frontal-subcortical networks [10,11], and frontal deterioration may lead to disinhibition of the parietal lobes, resulting in an increase in these hemodialyzed ESRD participants’ parietal lobe activation and a downward vertical bias. However, our results do not support this hypothesis.

Instead, our results indicate that, whereas people generally have an upward attentional bias while performing the vertical line bisection task, in the population of ESRD patients undergoing dialysis, the magnitude of this upward bias increases. In addition, the longer the duration of the illness, the larger the upward bias. In general, our findings have at least two possible explanations. The first is that there is disease-related deterioration of the dorsal visual stream, inducing an increase in the ventral stream relative to the dorsal stream, and this imbalance accounts for this increase in the upward bias. The second possibility is that the frontal-subcortical deficits lead to the disinhibition of the posterior temporal parietal lobes, and since the right hemisphere is dominant for both the leftward orienting of an attention and the upward orienting of visuo-spatial attention, not only do these hemodialyzed ESRD participants reveal an increased leftward attentional bias, as we have previously reported, but also an upward bias [2].

The visuospatial biases are considered a significant factor in increased risk of falling [32] and might lead to increased number of vehicle accidents [33]. Thus, there is a need to further investigate the visuospatial functioning of ESRD patients. Specifically, future studies should include a larger number of participants and using electrophysiological and imaging technology. Additionally, testing participants with ESRD who are not treated with dialysis might be useful to elucidate potential reason of the upward visuospatial bias (cerebrovascular vs. uremic intoxication origin). These future studies may allow us to better understand the neuropsychological mechanisms that account for these changes in the allocation of spatial attention and better develop means of correcting these attentional biases.

## 5. Conclusions

Alterations of spatial attention can have adverse effects, such as a greater probability of accidents. In this study ESRD patients receiving dialysis and healthy right-handed controls performed vertical line bisections. The hemodialyzed ESRD patients had a greater upward bias than healthy controls. Further, the magnitude of this bias was correlated with the duration of their kidney disease. The reason these patients have an increase in upward attentional bias is not known. Thus, future research is needed in order to better understand the brain mechanisms that might account for this bias as well as means that could possibly normalize alternations of spatial attentional in patients receiving dialysis due to ESRD.

## Figures and Tables

**Table 1:** Demographic, biochemical, and clinical data.

Variable	Group		
	Matched Controls (*n* = 23)	Dialyzed Patients (*n* = 23)	*p*-Value
Mean age ± SD	48.30 ± 14.82	46.78 ± 9.37	0.68
Mean years of education ± SD	15.04 ± 3.10	13.80 ± 2.38	0.78
MMSE ± SD	29.17 ± 0.78	28.43 ± 1.53	0.40
Anxiety ± SD	3.65 ± 2.76	3.35 ± 2.15	0.67
Depression ± SD	2.35 ± 2.15	3.43 ± 2.66	0.16
Sex (male), *n* (%)	13 (57)	14 (61)	0.76
Hypertension, *n* (%)	4 (17)	17 (85)	≤0.001 *
Primary kidney disease diagnosis, *n* (%)			
- Hypertensive nephropathy	NA	4	NA
- Glomerulonephritis	NA	4	NA
- Other	NA	15	NA
Mean duration of kidney disease (years) ± SD	NA	16.13 ± 15.14	NA
Mean time on dialysis (months) ± SD	NA	35.52 ± 24.37	NA
Mean blood urea nitrogen (BUN)(mg/dL) ± SD	13.95 (2.47)	30.87 ± 10.95	≤0.001 *
Mean creatinine (mg/dL) ± SD	0.82 (0.10)	8.00 ± 2.56	≤0.001 *
Mean albumin (g/L) ± SD	46.1 (1.78)	38.92 ± 3.28	≤0.001 *
Mean hemoglobin (g/dL) ± SD	14.32 (1.17)	11.62 ± 1.44	≤0.001 *

MMSE—mini-mental status examination; HADS—hospital anxiety and depression scale; Kt/V = kinetic transfer/volume; *—significant value.

**Table 2:** Correlation of medical–metabolic factors, disease duration, dialysis duration, and performance on vertical line bisection task.

Factor	Hemodialyzed Patients	Control Participants
Hemoglobin	r = 0.09 *p* = 0.71	r = −0.42 *p* = 0.06
Creatinine	r = −0.16 *p* = 0.45	r = 0.38 *p* = 0.10
Urea Nitrogen	r = −0.19 *p* = 0.41	r = 0.30 *p* = 0.91
Disease Duration	r = 0.51 *p* = 0.02 *	N/A
Dialysis Duration	r = −0.88 *p* = 0.71	N/A

*—significant result.
